# Pathomics in Gastrointestinal Tumors: Research Progress and Clinical Applications

**DOI:** 10.7759/cureus.85060

**Published:** 2025-05-29

**Authors:** Changming Lv, Yulian Wu

**Affiliations:** 1 Department of Surgery, The Fourth Affiliated Hospital, Zhejiang University School of Medicine, Yiwu, CHN; 2 Department of Surgery, The Second Affiliated Hospital, Zhejiang University School of Medicine, Hangzhou, CHN

**Keywords:** deep learning, gastrointestinal tumor, pathomics, prognosis prediction, whole slide images

## Abstract

Gastrointestinal tumors are among the malignancies with the highest global incidence and mortality rates, and their diagnosis and treatment heavily rely on histopathological examination. However, traditional pathological assessment faces challenges such as strong subjectivity, heavy workload, and low diagnostic consistency. In recent years, with advancements in high-resolution digital slide scanning technology and the rapid development of deep learning algorithms, pathomics has emerged as a novel tool for the precise diagnosis and treatment of gastrointestinal tumors. By extracting high-throughput quantitative features from whole slide images and combining machine learning and deep learning techniques, pathomics enables automated tumor typing, prognosis prediction, and treatment response evaluation. This article reviews the research progress of pathomics in gastrointestinal tumors, focusing on its applications in gene mutation prediction, prognosis assessment, and treatment response prediction, while analyzing current challenges and future directions.

## Introduction and background

Gastrointestinal tumors are among the malignancies with the highest global incidence and mortality rates. According to 2023 statistics from the United States, approximately 340,000 new cases of gastrointestinal tumors and 170,000 related deaths were reported [1,2]. The 2022 report from China’s National Cancer Center indicated that the overall incidence of gastrointestinal tumors reached 34%, with a cancer-related mortality rate as high as 44%. Among these, colorectal cancer ranked as the second most common malignancy in China, with approximately 510,000 new cases annually, while gastric cancer became the third leading cause of cancer-related deaths, following lung and liver cancers [3].

In clinical practice, histopathological examination of surgically resected tumor specimens serves as the gold standard for disease diagnosis and classification, typically performed by experienced pathologists. However, this process is highly labor-intensive, exhibits significant variability and subjectivity, and requires substantial time and effort. Additionally, the global shortage of pathologists and heavy clinical workloads raise concerns about diagnostic accuracy. Importantly, the vast amount of information contained in pathological images often exceeds the capabilities of human visual assessment. Therefore, there is a need for novel methods to assist pathologists in more efficient and rapid evaluation of pathological images while reducing subjectivity-related biases.

In clinical settings, histopathological staining techniques are primarily divided into three categories, namely, conventional histological staining, immunohistochemistry (IHC), and special staining. Among these, hematoxylin and eosin (H&E) staining, as a representative conventional method, is widely used for morphological assessment (e.g., cellular atypia) and histological grading of malignant tumors [4]. IHC staining enables the detection of specific proteins or antigens through antibody labeling, playing a critical role in tumor molecular subtyping, therapeutic target selection, and prognosis evaluation. Special staining provides supplementary diagnostic information for specific tissue components. Due to its cost-effectiveness, efficiency, and ability to provide comprehensive morphological information, H&E staining accounts for over 90% of basic pathological diagnostic scenarios.

With advancements in high-resolution slide scanning technology and reduced costs of digital storage, complete digitization of tissue sections has become feasible. The resulting whole slide images (WSIs) not only preserve histological architecture but also allow for further analysis of spatial structural features through machine learning. WSIs are more complex than many other imaging modalities, with large data sizes (single WSIs can reach 1 GB) and multi-scale image information depending on scanning magnification (20× or 40×). Furthermore, WSIs support multimodal analysis combining H&E and IHC staining, providing a rich data foundation for precision medicine.

The emergence of deep learning frameworks such as convolutional neural networks (CNNs) and graph neural networks (GNNs) has provided technical support for high-throughput analysis of WSIs. In this context, pathomics has emerged, utilizing advanced computational techniques to extract and quantitatively analyze features from pathological images [5]. Pathomics employs deep learning, image processing, and other machine learning techniques to perform detailed analysis of high-throughput image data from WSIs, including cellular morphology, spatial distribution, and tissue structure variations. Its applications encompass tumor identification, diagnostic classification, prognosis prediction, and treatment response evaluation, effectively advancing precision medicine.

## Review

WSI data acquisition and analysis workflow

As high-resolution digital pathological images, WSIs hold significant value in clinical diagnosis and medical research. However, due to large storage requirements and privacy concerns, publicly available WSI datasets remain limited. Notable open-access datasets include The Cancer Genome Atlas (TCGA) and the Clinical Proteomic Tumor Analysis Consortium (CPTAC).

**Figure 1:**
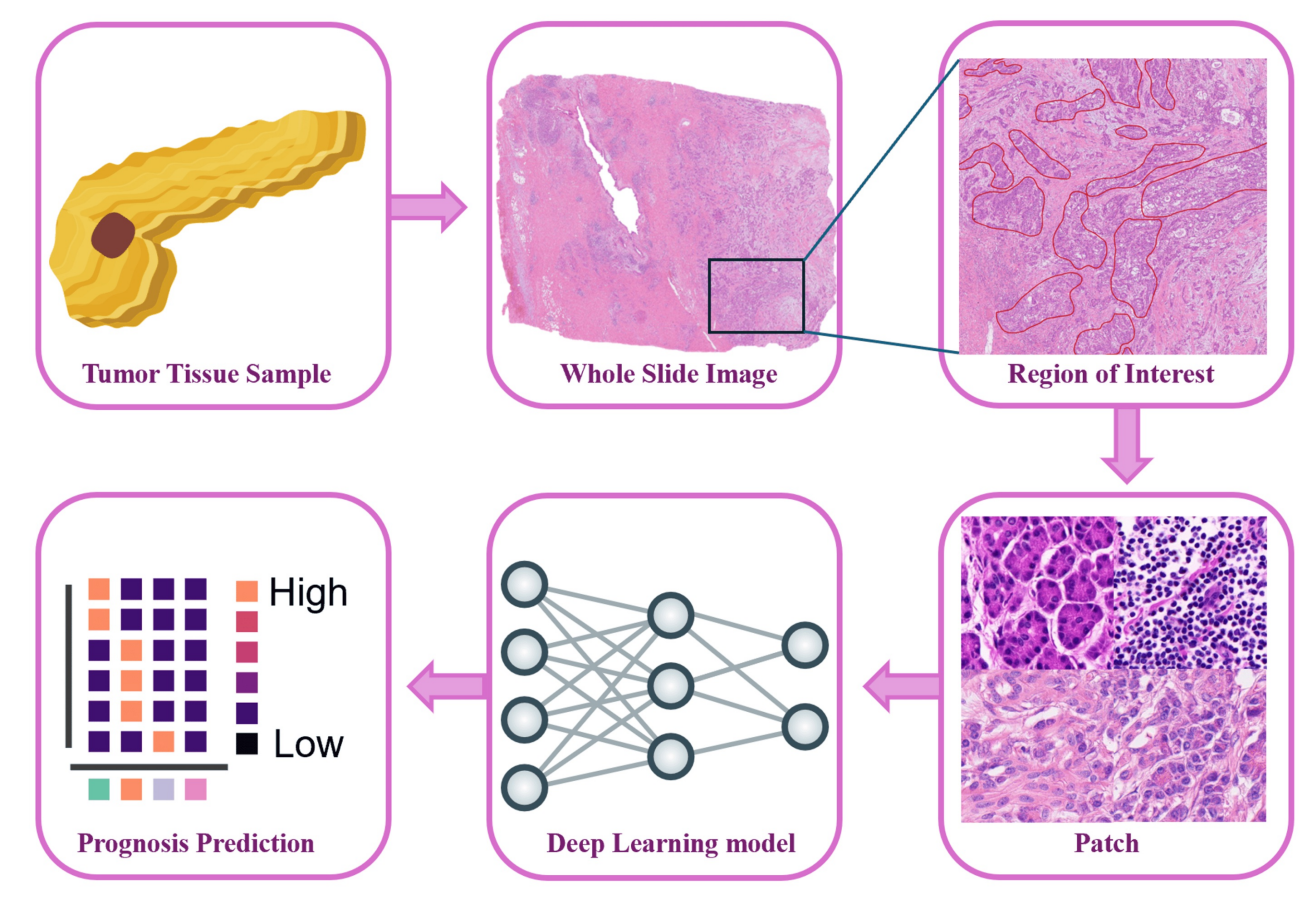
Framework of deep learning-based pathological image analysis pipeline. Image credits: This figure was created by the authors for this study.

Currently, there is no standardized framework for WSI processing, but the workflow typically includes the following key steps: image acquisition, quality control, annotation, color normalization, feature extraction and selection, and predictive model construction based on extracted features. Figure 1 illustrates the basic technical pipeline for WSI processing. This fundamental workflow has been widely adopted with various modifications across studies. For example, Tan et al. [6,7] implemented this framework to successfully predict pathological staging, while Qu et al. [8] applied a similar approach to develop a deep pathomics score for predicting hepatocellular carcinoma recurrence after liver transplantation. The specific implementation of these frameworks often varies according to research objectives, as demonstrated by Kludt et al [9], who employed multi-class tissue segmentation to identify prognosis-associated components and derive potential prognostic parameters.

The requirement for manual annotation remains particularly contentious in pathological image analysis due to its substantial labor intensity. Consequently, several studies [5] have explored weakly supervised learning paradigms for specific research applications, systematically comparing their performance against annotated approaches. Nevertheless, empirical studies confirm that comprehensive manual annotations achieve superior accuracy in most computational pathology applications, despite their substantial resource requirements.

WSI acquisition relies on high-precision digital scanners. Variability in scanner brands (e.g., Leica, Pannoramic), magnification (e.g., 20×, 40×), and staining protocols across institutions introduces heterogeneity in image quality and color distribution [10], potentially affecting analysis reliability. This heterogeneity is particularly pronounced in multi-center studies such as TCGA.

Quality control is essential to exclude WSIs or regions with poor image quality, artifacts, or contamination. Manual inspection or grayscale segmentation can identify non-compliant areas to ensure analysis accuracy.

Given WSI resolutions up to 100,000 × 100,000 pixels, direct processing of entire slides is computationally infeasible. Thus, WSIs are typically divided into smaller patches (e.g., 224 × 224 or 512 × 512 pixels) for analysis. WSIs contain diverse cell types (e.g., tumor cells, stromal cells, immune cells, adipocytes) and background regions. Pathologists often annotate or outline regions of interest (ROIs) based on research objectives. Annotation granularity ranges from case-level to cell-level and descriptive/multimodal annotations [11]. These annotations form the basis for training deep learning models. Recent advances in weakly supervised learning have reduced reliance on detailed annotations, enabling models to learn local features from slide-level labels [12].

To mitigate staining heterogeneity across WSIs, color normalization is a critical preprocessing step. Common methods include those reported by Macenko et al. [13], Vahadane et al. [14], Reinhard et al. [15], and Ruifrok et al. [16].

Following normalization, WSI patches undergo feature extraction. Traditional methods use tools such as CellProfiler [17,18] to extract low-level features (e.g., texture, granularity), while deep learning methods employ CNNs to automatically extract high-level features. Feature selection optimizes model performance by identifying the most discriminative features using statistical or machine learning techniques to reduce redundancy and enhance generalizability [19].

Based on extracted features, predictive models are constructed for tasks such as disease diagnosis, prognosis evaluation, and treatment response prediction. Common models include support vector machines, random forests, and deep learning models (e.g., ResNet, VGG). Model performance is typically evaluated via cross-validation or independent test sets.

Pathomics analysis techniques

WSIs contain vast biological information imperceptible to the human eye, requiring advanced analysis techniques for extraction and interpretation. Early pathomics relied on machine learning methods for quantitative analysis of image patches, which reduced computational complexity but inadequately captured complex biological information. With technological advancements, deep learning has become a standard tool in pathomics, significantly improving feature extraction precision and efficiency.

Traditional machine learning methods

Traditional machine learning methods have played a crucial role in feature extraction, selection, and model construction. Manual feature-based approaches primarily utilize software such as CellProfiler and QuPath to extract morphological (e.g., nuclear area, perimeter), texture (e.g., granularity), and color (e.g., color distribution) features. For example, several studies [19-21] used CellProfiler to extract morphological and texture features from image patches, successfully constructing prognostic models. Another study [22] employed QuPath for cell detection, extracting nuclear and cytoplasmic morphological features to build a machine learning-based pathomics prognostic model.

However, traditional machine learning methods in pathology analysis face significant constraints due to their reliance on manual ROI selection and localized processing. This approach introduces subjectivity through human-dependent ROI identification while systematically disregarding the vast majority of information contained in WSIs. By focusing analysis only on predetermined regions, these methods inherently lack both objectivity in feature selection and comprehensiveness in tissue evaluation, as critical diagnostic information outside selected ROIs and spatial relationships across the entire slide are overlooked [19]. These fundamental limitations in capturing the full complexity of pathological images have motivated the development of more advanced computational approaches.

Deep learning methods

Recent years have seen significant progress in deep learning applications for pathomics, particularly in image classification, object detection, and segmentation tasks.

CNNs are central to deep learning and are applied in the following three main areas: CNNs can automatically learn hierarchical features for tumor typing (e.g., adenocarcinoma vs. squamous carcinoma [23]) and grading (e.g., Gleason grading [24]); identify specific targets such as nuclei, lymphocytes, and adipocytes [25]; and delineate ROIs, such as tumor areas [26].

GNNs, designed for graph-structured data, capture spatial relationships between cells, enabling analysis of cellular interactions and spatial distributions within the tumor microenvironment (TME). A recent study [27] applied GNN-based models to characterize spatial tissue architecture at single-cell resolution, developing the first comprehensive clinical outcome prediction model for multiple diseases.

Transformers, originally developed for natural language processing, show promise in computer vision. They capture global dependencies in images and are suitable for large-scale WSI analysis, improving classification and segmentation accuracy through multi-scale feature integration.

While manual feature-based methods require close collaboration between pathologists and oncologists, deep neural networks automate feature learning through unsupervised approaches. Their performance depends on training data quality and annotation richness, with network designs prioritizing accuracy and computational efficiency [28]. However, manual feature-based methods offer superior interpretability, making them preferable for high-stakes decisions (e.g., prognosis or treatment benefit prediction), whereas deep learning excels in tasks such as object detection or segmentation where explainability is less critical.

Applications of pathomics

Pathomics provides a novel perspective and methodological support for disease prognosis assessment and treatment strategy formulation. This section will systematically explore three core applications of pathomics in gastrointestinal tumor research. First, it introduces the use of pathomics in predicting gene mutations, particularly focusing on advancements in deep learning models for predicting key genetic mutations from WSIs. Second, it discusses the application of pathomics in prognosis prediction, specifically how quantitative features of the TME can be utilized to construct prognostic models. Finally, it analyzes the potential of pathomics in predicting treatment responses, especially in immunotherapy and targeted therapy.

Gene Mutation Prediction

Genetic alterations in tumor cells can not only directly lead to functional changes that affect their morphological features but also remodel the TME, creating more complex genotype-phenotype correlations [10]. This higher-order relationship reflects the dynamic interactions between tumor cells and their surrounding environment. Conventional histopathological slides can intuitively capture these morphological changes, serving as a crucial foundation for related research.

Kather et al. [10] developed and optimized an end-to-end workflow based on tissue slide data from over 5,000 patients across 14 common solid tumor types in the TCGA database. This pipeline enables the prediction of gene mutations, molecular tumor subtypes, gene expression signatures, and standard pathological biomarkers from routine formalin-fixed, paraffin-embedded H&E-stained tissue sections. The study successfully identified numerous gene mutations significantly associated with detectable histological phenotypes, including key oncogenic pathway mutations such as *TP53*, *FBXW7*, *KRAS*, and *BRAF*, while further elucidating the complex links between genotype and phenotype. However, the results showed that for most molecular features, the predictive models achieved modest discriminative performance, with area under the receiver operating characteristic curve (AUC) values ranging between 0.6 and 0.7, indicating room for improvement in diagnostic performance. Future efforts should focus on training and optimizing models on larger datasets to enhance predictive accuracy and clinical utility.

Building on this research, Saldanha et al. [29] and Niehues et al. [30] further refined the analytical approaches, both adopting self-supervised feature extraction combined with attention-based Multiple Instance Learning (MIL). Saldanha et al., leveraging TCGA and CPTAC databases, demonstrated improved predictive performance in colorectal and pancreatic cancers, with external validation showing comparable results to the training set, highlighting strong generalization capability. Meanwhile, Niehues et al. achieved near-clinical-level performance in predicting microsatellite instability (MSI) status and *BRAF* mutations, though predictions for *KRAS*, *NRAS*, and *PIK3CA* mutations still fell short of clinical applicability, indicating a need for further model optimization. Both studies effectively addressed the limited generalizability observed in the study by Kather et al., robustly validating the superiority of self-supervised learning combined with MIL in digital pathology. This progress not only underscores the value of deep learning in tumor pathology image analysis but also provides new insights for developing more precise cancer diagnostic tools.

Further advancing the field, Guo et al. [31] developed a novel deep learning framework based on a Shifted Windows Transformer (Swin-T) backbone network for predicting MSI status and other key biomarkers in colorectal cancer. Their study demonstrated that the Swin-T-based architecture significantly improves predictive performance for MSI, *BRAF*, and other critical biomarkers, offering a new technical pathway for the continued advancement of digital pathology.

Prognosis Prediction

The TNM staging system is widely regarded as the cornerstone for prognostic prediction and treatment decision-making in gastrointestinal tumors. However, despite its critical clinical utility, the TNM system exhibits limitations in its ability to stratify patient prognosis, particularly due to the significant heterogeneity in outcomes and treatment responses observed among patients within the same stage.

Pathomics, as an emerging technological approach, enables the objective and precise quantification of key features within the TME, such as the spatial distribution of tumor cells, the tumor-to-stroma ratio, lymphocyte infiltration, and the extent of necrotic regions. These features reflect the biological behavior of tumors across multiple dimensions, ranging from tissue morphology to spatial organization, thereby providing more accurate prognostic information than TNM staging alone.

Chen et al. [19] analyzed WSIs from 480 gastric cancer patients, manually selected 10 representative image patches, and extracted pathomics features to construct a clinically applicable predictive model. This model not only served as a prognostic biomarker but also predicted patient responses to adjuvant chemotherapy. However, their method relied on manually extracted features, which introduced subjectivity and high time costs. In contrast, Zhao et al. [26] trained a CNN on publicly available datasets to perform a nine-class classification task on colorectal cancer WSIs, including categories such as adipose tissue, background, necrosis, lymphocytes, mucus, smooth muscle, normal mucosa, stroma, and tumor epithelium. The model achieved an impressive classification accuracy of 97.46% on the test set. Notably, the study identified the model-predicted tumor-stroma ratio (TSR) as an independent prognostic factor for overall survival (OS), with lower TSR significantly associated with poorer outcomes. This research demonstrated the feasibility of fully automated TSR scoring from H&E-stained WSIs, further validating the potential of pathomics in prognostic assessment. Building on this, Shi et al. [32] employed a deep learning model to automatically quantify the composition of the TME in colorectal cancer and developed a prognostic model based on TME features. Using a VGG19-based architecture, the team successfully identified the nine aforementioned cell types, with seven defined as TME components (excluding background and necrosis). By analyzing WSIs and survival data from the TCGA colorectal cancer cohort, they constructed a TME feature model significantly correlated with progression-free survival. Subsequent gene enrichment analysis elucidated relevant signaling pathways, offering novel biological insights into the role of the TME in colorectal cancer progression.

Treatment Response Prediction

Approximately 15% of gastric and colorectal cancer patients benefit from immune checkpoint inhibitor therapy [33,34]. MSI is a key biomarker for predicting immunotherapy response in gastrointestinal tumors [35]. However, in clinical practice, MSI status typically requires confirmation through additional tests such as gene sequencing or IHC.

Kather et al. [36] developed a deep learning-based MSI prediction model by analyzing over 1,000 WSIs from more than 400 gastric and colorectal cancer patients in the TCGA database. The team first employed ResNet18, a lightweight yet highly accurate deep learning model, to construct a three-class classifier (tumor cells, stromal cells, and adipocytes), achieving efficient tumor detection with an AUC exceeding 0.99. They then used another ResNet18 model to distinguish between MSI and microsatellite stability, attaining an AUC of 0.81 for MSI detection in gastric cancer, with similar performance (AUC = 0.84) in an external validation set. This study pioneered the direct prediction of MSI status from H&E-stained tissue slides using deep learning, offering a novel technical approach for more accurate MSI patient identification.

Despite the importance of MSI as an immunotherapy biomarker, its predictive efficacy remains limited. To improve immunotherapy response prediction, a multi-center study developed a pathomics-driven ensemble model using H&E-stained WSIs and clinical data from 584 gastric cancer patients. The model demonstrated excellent predictive performance in both training and validation cohorts. To elucidate its biological mechanisms, the team conducted pathogenomic analyses, revealing associations between model predictions and immune-, cancer-, and metabolism-related pathways [37]. For advanced gastric cancer, Liu et al. [38] introduced the Immune Checkpoint Inhibitors Response Score, a novel histopathological biomarker that effectively predicts responses to programmed cell death-1 inhibitor combined with chemotherapy, providing a new tool for personalized treatment planning.

Furthermore, in gastric cancer, HER2 positivity defines an important subtype, with HER2-targeted immunotherapy significantly improving outcomes in advanced cases. However, current HER2 protein assessment in IHC relies heavily on manual evaluation. Pathologists typically perform semi-quantitative scoring under the microscope, repeatedly comparing H&E- and IHC-stained WSIs while adjusting magnification to identify suspicious cancerous regions, ultimately classifying them into four grades (0, 1+, 2+, and 3+). To refine this process, Han et al. [39] developed an automated HER2 scoring framework for gastric cancer by first annotating image patches according to IHC scoring guidelines and then training a deep learning model to score entire WSIs automatically. This framework not only enhanced scoring efficiency but also reduced interobserver variability, offering technical support for precise HER2-targeted therapy implementation.

Challenges and future perspectives

While pathomics holds transformative potential for precision oncology, several critical challenges must be addressed to realize its clinical adoption. A fundamental limitation lies in annotation dependence, where current methodologies require labor-intensive manual annotations (consuming hours per WSI) while demonstrating suboptimal interpathologist consistency (kappa <0.8) [40], compounded by tumor-specific morphological patterns that restrict cross-cancer generalization and demand large annotated datasets for each malignancy. However, a pivotal pathomics advancement involves adopting weakly/self-supervised learning methods such as MIL to minimize annotation dependency. MIL utilizes only slide-level labels while automatically detecting diagnostically relevant regions, bypassing labor-intensive, pixel-level annotations. When integrated with self-supervised pretraining, this approach significantly reduces labeling efforts while preserving high diagnostic performance. This paradigm shift addresses critical implementation barriers in digital pathology, dramatically cutting annotation time, mitigating interobserver variability, and facilitating broader clinical adoption of artificial intelligence (AI)-powered pathological analysis across healthcare systems. The technique demonstrates particular promise for resource-constrained settings and rare disease research.

Beyond annotation challenges, data heterogeneity across institutions, manifested through scanner variability and staining protocol differences, undermines reproducibility despite existing normalization techniques (e.g., Macenko/Vahadane methods), with the field still lacking globally accepted standardization protocols. Perhaps most critically, the interpretability barrier persists as black-box models obscure decision logic; while prognostic features such as stromal fibrosis show empirical correlations with outcomes, their underlying biological mechanisms (such as relationships with immune checkpoint expression profiles) remain mechanistically unclear. These technical hurdles intersect with growing data privacy concerns, where stringent regulations limit WSI sharing and create reliance on limited public datasets (TCGA/CPTAC) that risk model overfitting, a situation exacerbated by institutional data silos that impede independent validation studies.

Additionally, the integration of pathomics with genomics, radiomics, and clinical data represents a transformative future direction for precision medicine. By combining these multimodal datasets, researchers can uncover deeper insights into disease mechanisms, improve diagnostic accuracy, and personalize treatment strategies. For instance, pathomics can reveal tumor morphology, while genomics provides molecular alterations, together enhancing cancer subtyping and prognostic predictions. Radiomics adds imaging-based phenotypic data, offering non-invasive longitudinal monitoring, and clinical data contextualizes findings for real-world applicability. However, challenges remain, including data standardization, interoperability, and the need for advanced AI algorithms to integrate heterogeneous datasets. Future efforts should focus on developing unified frameworks for multimodal data fusion, ensuring robust validation across diverse cohorts. Additionally, interdisciplinary collaboration among pathologists, radiologists, geneticists, and clinicians will be critical to translate these insights into clinical practice.

In the long term, this integration could enable dynamic, patient-specific disease profiling, guiding targeted therapies and improving outcomes. The future of pathomics lies not in isolation but as a pivotal component of a holistic, data-driven healthcare paradigm. Thus, expanding this synergy should be a priority in forthcoming research to realize its full potential in precision medicine.

Collectively, these challenges frame the essential roadmap for future research: developing annotation-efficient learning paradigms, establishing universal preanalytical standards, advancing explainable AI architectures, creating federated learning frameworks that reconcile data utility with privacy preservation, and achieving multi-omics integration.

Future directions

Key future directions for pathomics include adopting weak/self-supervised learning, such as MIL, to reduce annotation needs using slide-level labels; multimodal integration combining pathomics with genomics, radiomics, and clinical data [6,41]; standardization of scanning (40×, 0.25 µm/pixel), staining, and quality control protocols; developing explainable AI using attention mechanisms to visualize critical regions validated against known biomarkers; and implementing privacy-preserving federated learning for secure multi-center collaborations.

## Conclusions

Pathomics, bridging digital pathology and AI, is transforming gastrointestinal oncology. By quantifying WSI features (morphology, spatial architecture, TME), it surpasses TNM staging in prognosis and therapy prediction (e.g., MSI/HER2 models). Challenges, e.g., annotation bottlenecks, data variability, and model opacity, require solutions such as standardization, explainable AI, and privacy-aware technologies. Future integration with multi-omics will solidify the role of pathomics in precision medicine.
